# Establishment of a Genetically Confirmed Breeding Colony of *Mastomys natalensis* from Wild-Caught Founders from West Africa

**DOI:** 10.3390/v13040590

**Published:** 2021-03-31

**Authors:** David Safronetz, Kyle Rosenke, Robert J. Fischer, Rachel A. LaCasse, Dana P. Scott, Greg Saturday, Patrick W. Hanley, Ousmane Maiga, Nafomon Sogoba, Tom G. Schwan, Heinz Feldmann

**Affiliations:** 1 Laboratory of Virology, Rocky Mountain Laboratories, National Institute of Allergy and Infectious Diseases, National Institutes of Health, Hamilton, MT 59840, USA; kyle.rosenke@nih.gov; 2 Laboratory of Zoonotic Pathogens, Rocky Mountain Laboratories, National Institute of Allergy and Infectious Diseases, National Institutes of Health, Hamilton, MT 59840, USA; fischerro@niaid.nih.gov (R.J.F.); tom.schwan@nih.gov (T.G.S.); 3 Rocky Mountain Veterinary Branch, Rocky Mountain Laboratories, National Institute of Allergy and Infectious Diseases, National Institutes of Health, Hamilton, MT 59840, USA; rlacasse@niaid.nih.gov (R.A.L.); dana.scott@nih.gov (D.P.S.); greg.saturday@nih.gov (G.S.); patrick.hanley@nih.gov (P.W.H.); 4 International Center for Excellence in Research (ICER-Mali), Faculty of Medicine and Odonto Stomatology, University of Sciences, Techniques and Technologies of Bamako (USTTB), Bamako, Mali; ousmanem@icer.org (O.M.); nafomon@icer.org (N.S.)

**Keywords:** *Mastomys natalensis*, natal multimammate mouse, natal multimammate rat, soft-furred african mouse, african rat, rodent breeding colony, lassa virus

## Abstract

*Mastomys natalensis* are a ubiquitous and often dominant rodent across sub-Saharan Africa. Importantly, they are a natural reservoir for microbial pathogens including Lassa virus (LASV), the etiological agent of Lassa fever in humans. Lassa-infected rodents have been documented across West Africa and coincide with regions where annual outbreaks occur. Zoonotic transmission to humans most often occurs directly from infected rodents. Little is known about LASV infection kinetics and transmissibility in *M.* *natalensis*, primarily due to available animals. Here, we describe the establishment of a laboratory breeding colony of genetically confirmed *M.* *natalensis* from wild-captured rodents. This colony will provide a convenient source of animals to study LASV and other emerging pathogens that utilize *M. natalensis* in their enzootic lifecycles.

## 1. Introduction

The genus *Mastomys* (family Muridae) currently contains seven or eight species of rodents [1]. The taxonomic history of *Mastomys* rodents is somewhat complicated. Based on appearance, they have been referred to as rats, mice, and *Praomys*, a closely related African rodent, earlier in taxonomic reports [1]. The advent of molecular techniques has recently determined that *Mastomys* is a unique genus within the Muridae family and remains a close relative of *Praomys* spp. rodents [1].

**Figure 1:**
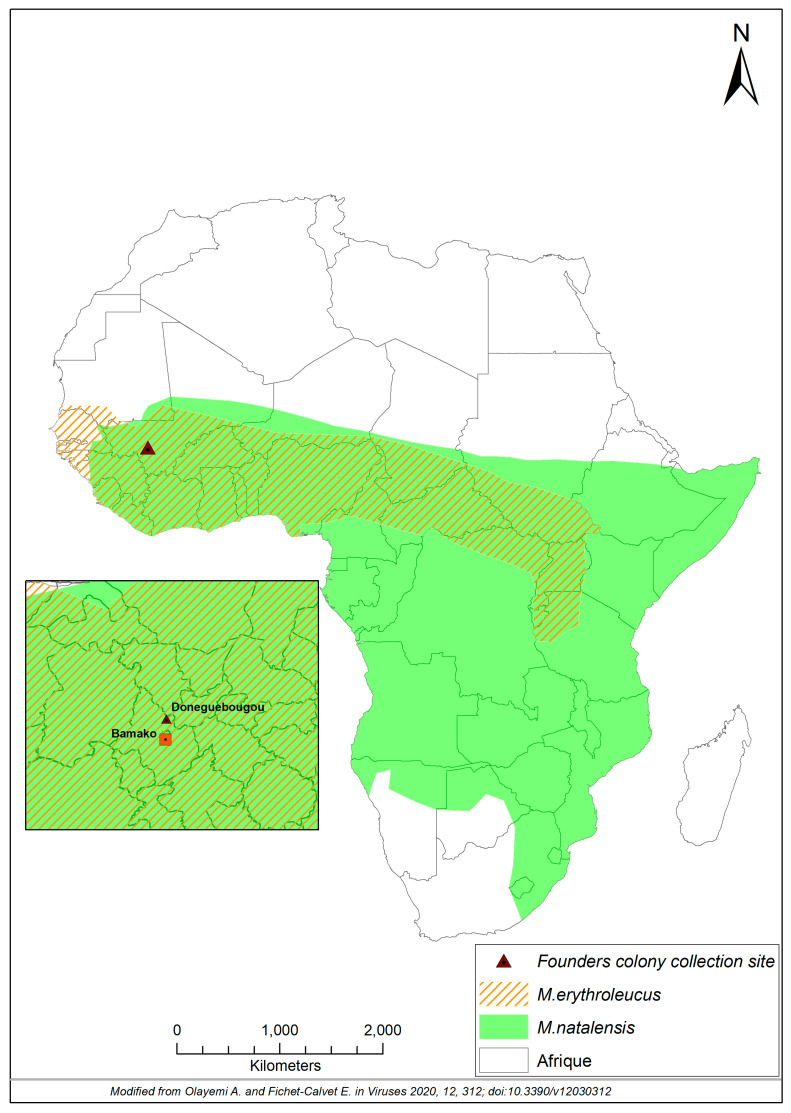
Geographic distribution of *Mastomys natalensis* and *Mastomys erythroleucus* in sub-Saharan Africa. Shown are the distributions of *M. natalensis* (shaded green) and *M. erythroleucus* (red-hatched) across sub-Saharan Africa. Inset denotes the village of Doneguebougou (triangle), where the founder rodents for the colony were live-trapped, in relation to the capital city of Bamako, Mali.

**Figure 2:**
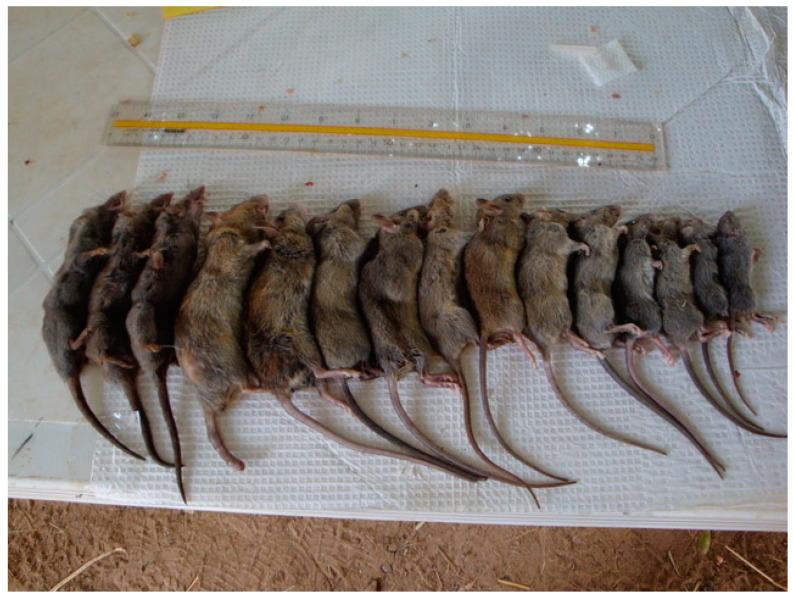
Wild-captured *Mastomys natalensis* in Mali. The image shows the differing physical appearances of *M. natalensis* captures in Mali, in relation to the relatively uniform giant shrew (three animals on the left side of the image).

*Mastomys* rodents are ubiquitous across sub-Saharan Africa, many of which live sympatrically and are nearly indistinguishable by appearance alone (Figure 1 and Figure 2). For example, the adult pelage of *M. natalensis* in Mali varied greatly from a lighter sandy brown to extreme melanistic forms that are nearly black. Further, the bellies of *M. natalensis* tended to be darker in color than that of *M. erythroleucus*, which were more frequently white [2] (Tom Schwan, unpublished observations). A common trait of *Mastomys* rodents is a 1:1 ratio in body to tail length. In many areas *Mastomys* are the predominant mammalian species. On average, pups weighted 10–14 g while adult animals averaged approximately 60 g (ranging between 30 and 108 g, Tom Schwan unpublished data). Their omnivorous and commensal habits put them in direct contact with human populations, making them an important rodent species to study as a rodent reservoir for emerging pathogens.

Approximately 50 years ago, Lassa virus (LASV; *Arenaviridae*, genus *Mammarenavirus*) was discovered on the Jos plateau in Nigeria [3]. Its association with *M. natalensis* was established as was the endemic region (West Africa) where infections in humans were occurring annually [4]. Over the last several decades, numerous field and ecological studies have been conducted on LASV, mostly focusing on prevalence of infection using molecular (RT-PCR) or serological (ELISA) techniques [5]. To date, little emphasis has been placed on studying the virus/rodent host interactions, due in large part to a lack of colonized *M. natalensis*. Although *M. natalensis* rodents have been colonized and utilized as feeder rats for herpetologists, genetic analyses have shown these animals are *M. coucha.* While these animals have shown utility for certain cancer, autoimmune, and infectious disease models [6,7,8,9,10], their use in studying LASV dynamics remains minimal.

The lone attempt to understand LASV infection kinetics in an appropriate rodent host was conducted by Walker et al. [11], in which they described infection of *Mastomys* rodents with an undefined dose or source of LASV. However, they did not determine if the wild-captured rodents were *M. natalensis,* which can have a variety of physical appearances (Figure 2) or sympatric and the nearly morphologically identical *M. erythroleucus* or *M. huberti.*

The objective of this study was to establish a genetically confirmed laboratory colony of *M. natalensis* for use in subsequent experiments aimed at studying LASV/rodent host interactions as well as other emerging pathogens from West Africa.

## 2. Materials and Methods

### 2.1. Trapping and Quarantine of Founder Stock

The founders of the *M. natalensis* colony were live-trapped in peridomestic settings around Doneguebougou (12°48′21″ N–7°59′0″ W), a rural village in southern Mali, approximately a 1 h drive from Bamako (Figure 1). This site was selected for collection of founder stock as our previous studies had shown a high prevalence of *M*. *natalensis* with a low diversity of other rodent species in Doneguebougou [5]. Furthermore, we have not detected a single LASV positive rodent in this village (by RT-PCR or serology) suggesting the virus was not present in this region [5]. Over the course of three consecutive nights in 2012, Sherman live traps were baited with a mixture of onions and crushed peanuts and set out in the late afternoon in areas of known rodent activity. The following morning, traps were collected to process the captured animals. To prevent escape or injury, captured animals were anesthetized by inhalational isoflurane before removal from traps and then assessed for inclusion as founder stocks. Animals that were visually identified as *Mastomys* spp. rodents and were sub-adult in good body condition (no signs of existing or previous wounds) were kept to establish the colony; all others were sampled as a part of ongoing surveillance programs and humanly euthanized. Sampling included the collection of a blood sample and a small punch biopsy from the ear pinnae for cytochrome b sequence analysis as previously described [12,13]. Given the well-developed musculature, saphenous bleeds were not effective; therefore, blood was collected via the intra-orbital sinus. Pertinent biological information including gender, weight, and body and tail length was also recorded. Potential founders were dusted with permethrin to remove ectoparasites and given a subcutaneous injection of Ivermectin (0.2 mg/kg) to treat parasite infections.

Potential founders were transported to the University of Sciences, Techniques and Technologies of Bamako Medical campus located at Point G and maintained for 3–5 days in static filtered caging units, after which they were transferred to a small animal breeding facility in Bamako (Centre National d’Appui à la lutte contre la Maladie, CNAM). Blood samples were repeatedly collected from all animals for routine molecular and serological LASV testing.

### 2.2. Transfer of Animals to Rocky Mountain Laboratories

The founder stock of the Rocky Mountain Laboratories (RML) colony was transferred from Bamako, Mali to RML in Hamilton Montana, according to national and international (IATA) regulations. Export out of Mali required a veterinary health certificate provided by a licensed veterinarian in Mali and a Malian export permit. Entry into the United States required permits from the Centers for Disease Control and Prevention and a Montana State exotic animal agreement, as well as a United States Fish and Wildlife Service inspection. All permits were issued in 2013 and are available upon request.

### 2.3. Animal Husbandry

**Figure 3:**
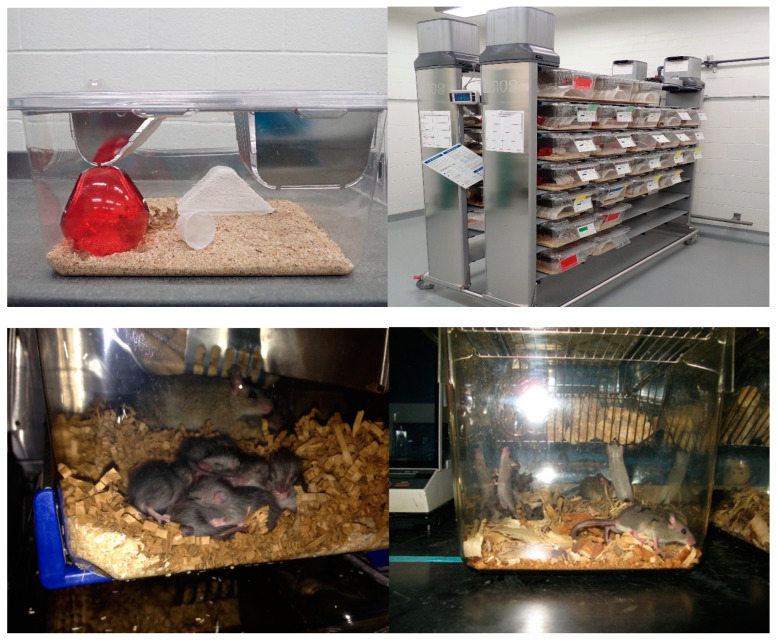
Caging setup for the *Mastomys natalensis* colony. Upper left: standard disposable caging unit with “locking” wire top, bedding, and enrichment. Upper right: ventilated rack dedicated to the *Mastomys natalensis* colony. Bottom left: dam and pups in a ventilated cage. Bottom right: sub adult *Mastomys natalensis* in a static caging unit in Mali just prior to shipment to Rocky Mountain Laboratories (RML).

After importation, *M. natalensis* were maintained in breeding pairs in individually ventilated cages (Figure 3). Animals were kept in a dedicated room at a 12 h light cycle, 22 ± 2 °C, and 40–60% relative humidity in individually ventilated disposable cages (Innocage^®^ IVC Rat Caging System, Innovive, San Diego, CA, USA), with autoclaved bedding (Sani-Chips^®^, P.J. Murphy Forest Product Corp., Montville, NJ, USA), and provided with ab libitum rodent chow (2018 Teklad global 18% protein rodent diet, Envigo Teklad, Denver, CO, USA) and reverse osmosis water. Enrichment included nesting material (Enviro-dri^®^, Shepherd Specialty Papers, Watertown, TN, USA) and shelters (Shepherd Shack^®^, Shepherd Specialty Papers, Watertown, TN and/or Crawl Balls^TM^, Bio-Serve^®^, Flemington, NJ, USA). For the first several months in captivity, animals were lightly sedated weekly with inhalational isoflurane while in their cages for health inspections and cage changing.

### 2.4. Sentinel Testing of Colony Animals

Sentinel testing at RML was conducted using standard dirty-bedding sentinels. Briefly, following a cage change, pathogen-free ICR mice were exposed to the dirty bedding for 3–4 weeks. Post-exposure, serum samples were collected from sentinel mice and submitted to an external laboratory for murine pathogen testing. Pathogens tested for included: ectromelia virus, mouse rotavirus, lymphocytic choriomeningitis virus, mouse adenovirus, Sendai virus, mouse hepatitis virus, minute mouse virus, mouse parvovirus, mouse polyoma virus, mouse norovirus, Theiler’s murine encephalomyelitis virus, *Mycoplasma pulmonis*, pinworms, and ectoparasites.

Initially, sentinel testing was conducted frequently; however, after the colony was established it was reduced to four times per annum or immediately prior to a large experiment. Because of the uncertainty of potentially biased negative results due to host susceptibility of ICR mice to endogenous *M. natalensis* pathogens, random animals from the colony were selected, euthanized, and a complete necropsy and examination was conducted by a certified veterinary pathologist to look for potential signs of infection or disease.

### 2.5. Susceptibility to LASV Infection

To initially assess the susceptibility of the lab-reared *M. natalensis* to infection with LASV, a pilot study was conducted consisting of a single group of six rodents (three males, three females, aged 2–6 months) inoculated with 8.3 × 10^3^ 50% tissue culture infectious doses (TCID_50_) of LASV Soromba-R via the intraperitoneal (i.p.) route [14]. On day 10 post-infection, the animals were euthanized and tissue samples (blood, liver, lung, spleen, kidney, bladder, heart, and brain), as well as oral and rectal swabs, and, when available, urine, were collected for virological analysis. Samples were inactivated, extracted, and tested for the presence of LASV RNA using previously described one-step, non-strand specific, real-time RT-PCR methods [5]. All animal experiments were approved by the Institutional Animal Care and Use Committee of Rocky Mountain Laboratories (document number 2014–001), NIH and carried out by certified staff in an Association for Assessment and Accreditation of Laboratory Animal Care (AAALAC) International accredited facility, according to the institution’s guidelines for animal use, following the guidelines and basic principles in the NIH Guide for the Care and Use of Laboratory Animals, the Animal Welfare Act, United States Department of Agriculture and the United States Public Health Service Policy on Humane Care and Use of Laboratory Animals. All work with infectious LASV and potentially infectious materials derived from animals was conducted in a Biosafety Level 4 (BSL 4) laboratory in the Integrated Research Facility of the Rocky Mountain Laboratories (RML), National Institute of Allergy and Infectious Diseases (NIAID), NIH. Sample inactivation and removal was performed according to standard operating protocols approved by the local Institutional Biosafety Committee.

## 3. Results

### 3.1. Quarantine Testing and Transfer

Over the course of 4 weeks in Bamako, founder animals repeatedly tested negative for LASV infection by serological and molecular methodologies. Genetic testing (cytochrome b sequence analysis) confirmed all 20 captive rodents (10 male, 10 female) were *M. natalensis.* The founder stock was intended to be shipped to RML approximately 2 months after field collections; however, due to sudden political instability in Mali, the shipment was significantly delayed resulting in litters born in Mali. At the time of transfer, approximately 18 months after the original breeders were captured, a total of 90 rodents were in captivity in Mali and shipped to RML according to US federal and international regulations to expand the colony and for subsequent experiments. Based on the repeated negative test results for LASV, in addition to consistent negative results for LASV in previous rodents collected in the community where the founders were trapped, no further LASV testing was conducted prior to shipment to RML.

### 3.2. Sentinel Testing

Serological testing did not find evidence of ectromelia virus, mouse rotavirus, lymphocytic choriomeningitis virus, mouse adenovirus, Sendai virus, mouse hepatitis virus, minute mouse virus, mouse parvovirus, mouse polyoma virus, mouse norovirus, Theiler’s murine encephalomyelitis virus, or *Mycoplasma pulmonis*. Additionally, no evidence of pinworms was noted. If the presence of ectoparasites was suspected, food supplemented with Ivermectin replaced regular mouse chow. Necropsies performed by a certified veterinary pathologist on a small subset of rodents, both directly shipped from Mali as well as laboratory-reared animals from RML, did not document any irregularities.

### 3.3. Temperament

Not surprisingly, the *M. natalensis* proved to be more difficult to handle than routine laboratory rodents. Due to their speed and agility, strict protocols were established to handle these animals, which included sealing off the room to prevent escape or loss and lightly sedating animals while in their cages with inhalational isoflurane prior to cage changes and hands-on health checks. Additionally, although the rodents were not overly aggressive with animal caretakers, bite-proof Kevlar gloves were worn whenever handling the rodents, even when under sedation.

Aggressive behavior between rodents was not common, though it should be noted that non-litter mates of the same sex were not socially housed together. Overall, the majority of aggressive events observed were in breeding pairs and most commonly consisted of female aggression toward potential or established male suitors. Cannibalism was also infrequent with only three incidents, two of which were stillborn births, observed during the first 12 months of breeding at RML.

### 3.4. Reproductive Behavior

Due to the delay in shipping the founder stock, 10 breeding pairs were set up from the original 20 rodents. In total, 90% of the initial pairs were fertile, resulting in the 90 rodents that were transferred to RML. Over the course of the first 12 months of the RML *M. natalensis* breeding program, 166 litters were born comprising 1618 (755 females, 863 males) pups. The average litter size was 9 pups, with a range of 2 to 16 per litter. In general, smaller litters were noted in the first pregnancy of younger breeders, with subsequent litters from these animals tending to trend towards the colony average. Compared with other rodent colonies maintained at RML, *M. natalensis* tended to reach sexual maturity and breeding potential later in life, on average around 4 months of age. Courting time for breeding pairs varied as well, with approximately one third of pairs mating almost immediately, while two-thirds taking up to 8 weeks before pregnancy was achieved. Nevertheless, the overwhelming majority of breeding pairs set up at RML (>98%) resulted in offspring. Overall, there was a very low incidence of stillbirths, with only two of the 166 litters in the first 12 months containing small numbers of dead pups. Both events occurred in the first litter of new (younger) breeders and no subsequent issues were noted in either breeding pair.

Both parents participated in the care of the pups. The dam did the majority of the work; however, the sire cared for the young while the dam fed and groomed. The preference for the dam and sire to remain together while raising the young often led to secondary litters being born while previous litters were still being cared for. These so called “litters on litters” were common, though they did not appear to be a problem for the parental units to maintain. In general, the sire occupied the older pups while the dam nursed the infant pups. Due to this phenomenon, colony-reared pups tended to be weaned between 16 and 20 days old since the average gestation period was estimated at 19–21 days.

While not addressed in the RML colony breeding documents, there appeared to be no adverse effects on the number of pregnancies or the number of pups per pregnancy associated with the age of the dam. Similarly, longevity and life span of the animals were not addressed in these studies, although rodents exceeding 1.5 years in captivity were documented.

### 3.5. Susceptibility to LASV Infection

Experimental infection of *M. natalensis* had no effect on body weight during the 10-day pilot study. The majority of organ specimens collected at 10 dpi from experimentally infected animals demonstrated readily detectable levels of LASV RNA (Table 1). In addition, oral and rectal swabs from all six animals were positive for the presence of LASV RNA. Urine (collected via cystocentesis) was only available from two animals, both of which were LASV RNA positive (Table 1). LASV was re-isolated from a subset of tissues using standard tissue culture methodologies, thereby confirming the presence of infectious virus in these animals.

## 4. Discussion

Approximately 60% of emerging pathogens are of zoonotic origin and, given the diversity of the order Rodentia (≥2400 species including over 1600 murid rodents) [15], many of these pathogens use rodent reservoirs as ecological hosts [1,16]. Despite this, little effort has been placed on studying the virus/rodent host interface in a controlled laboratory environment. The reasons for this are many and include a lack of host-specific reagents and deficiencies in funding opportunities. However, the main impediment is that few colonies for meaningful studies on selected pathogens exist. Yet, once established, the data gleaned from these colonies can help shape our understanding of pathogenesis of human diseases. Shortly after the discovery of hantavirus pulmonary syndrome (HPS) and Sin Nombre virus (SNV), scientists at the University of New Mexico established a small colony of deer mice, *Peromyscus maniculatus*, the natural rodent reservoir of SNV [17]. The *Peromyscus* colony not only allowed for the study of viral kinetics and pathogenicity (more specifically a lack thereof) but was also imperative in the formulation of a leading theory on the pathogenesis of SNV in humans, specifically a lack of T regulatory cell responses in cases of HPS compared to strong T regulatory responses in deer mice [18,19]. The colony has also lent itself to understanding mutations acquired in SNV when lab-passaged in standard mammalian cell cultures, efforts in developing potential vaccines, and proved to be the missing piece in the development of a non-human primate model for HPS [20]. All of these innovations stemmed from a small group of scientists with the foresight to establish a colony.

Here we sought to do similar work, albeit with an African rodent species, for the study of LASV. The colony is now firmly established and has already proved useful in studies on the ecological role *M. natalensis* play in the maintenance cycle of an African relapsing fever spirochete [21]. The pilot studies above have demonstrated the colony-reared *M. natalensis* are susceptible to LASV infection with systemic spread of the virus. Additionally, positive viral swabs suggest the utility of this colony for studying transmission of LASV directly from rodent reservoirs. On-going efforts are now focused on long-term LASV/host infection dynamics and transmission. Given the prolific breeding potential of the laboratory colony, it is well suited to expand and facilitate several studies per year, not only on LASV but also on other potential rodent-borne human pathogens of concern from across sub-Saharan Africa.

## Figures and Tables

**Table 1:** Qualitative molecular analysis of tissues collected at 10 days post-infection from laboratory-reared *Mastomys natalensis* with Lassa virus.

Sample	No. Positive */No. Tested
Lung	6/6
Liver	6/6
Spleen	6/6
Heart	6/6
Kidney	5/6
Bladder	6/6
Brain	6/6
Blood	5/6
Oral swab	4/6
Rectal swab	3/6
Urine	2/2

* Sample determined to be positive if Ct was less than 32.
